# Preliminary investigation of the diagnosis and gene function of deep learning PTPN11 gene mutation syndrome deafness

**DOI:** 10.3389/fgene.2023.1113095

**Published:** 2023-01-25

**Authors:** Xionghui Wu, Min Huang, Weiqing Huang, Sijun Zhao, Jiang Xie, Guangliang Liu, Shuting Chang

**Affiliations:** ^1^ Department of Otorhinolaryngology Head and Neck Surgery, Hunan Children’s Hospital, Changsha, Hunan, China; ^2^ Department of Neonatology, Hunan Children’s Hospital, Changsha, Hunan, China

**Keywords:** syndrome deafness, deep learning, intelligent medicine, PTPN11 gene mutation, diagnosis and gene function

## Abstract

Syndromic deafness caused by PTPN11 gene mutation has gradually come into the public’s view. In the past, many people did not understand its application mechanism and role and only focused on non-syndromic deafness, so the research on syndromic deafness is not in-depth and there is a large degree of lack of research in this area. In order to let the public know more about the diagnosis and gene function of deafness caused by PTPN11 gene mutation syndrome, this paper used deep learning technology to study the diagnosis and gene function of deafness caused by syndrome with the concept of intelligent medical treatment, and finally drew a feasible conclusion. This paper provided a theoretical and practical basis for the diagnosis of deafness caused by PTPN11 gene mutation syndrome and the study of gene function. This paper made a retrospective analysis of the clinical data of 85 deaf children who visited Hunan Children’s Hospital,P.R. China from January 2020 to December 2021. The conclusion were as follows: Children aged 1–6 years old had multiple syndrome deafness, while children under 1 year old and children aged 6–12 years old had relatively low probability of complex deafness; girls were not easy to have comprehensive deafness, but there was no specific basis to prove that the occurrence of comprehensive deafness was necessarily related to gender; the hearing loss of patients with Noonan Syndrome was mainly characterized by moderate and severe damage and abnormal inner ear and auditory nerve; most of the mutation genes in children were located in Exon1 and Exon3, with a total probability of 57.65%. In the course of the experiment, it was found that deep learning was effective in the diagnosis of deafness with PTPN11 gene mutation syndrome. This technology could be applied to medical diagnosis to facilitate the diagnosis and treatment of more patients with deafness with syndrome. Intelligent medical treatment was also becoming a hot topic nowadays. By using this concept to analyze and study the pathological characteristics of deafness caused by PTPN11 gene mutation syndrome, it not only promoted patients to find diseases in time, but also helped doctors to diagnose and treat such diseases, which was of great significance to patients and doctors. The study of PTPN11 gene mutation syndrome deafness was also of great significance in genetics. The analysis of its genes not only enriched the gene pool, but also provided reference for future research.

## 1 Introduction

The diagnosis of deafness in PTPN11 gene mutation syndrome has not been studied too deeply at present. Some scholars only analyzed non-comprehensive deafness, but they did not analyze the situation of comprehensive deafness, which also led to that people were not familiar with syndrome deafness and did not often pay attention to it. In order to remedy this defect, it is of practical significance to study the diagnosis and gene function of deafness caused by PTPN11 gene mutation syndrome.

Deafness is a multiple disease, which has been studied by some scholars. Xu H Y analyzed the relationship between PTPN11 gene and deafness (Xu et al., 2019). Gao Y analyzed the clinical phenotype and gene of syndromic deafness with PTPN11 gene mutation (Gao et al., 2022). GaoXue believed that congenital sensorineural hearing loss was the initial manifestation of PTPN11 related NS, which was accompanied by multiple spots or NS (Gao et al., 2021). Huang S S analyzed the case report and diagnosis of multi spot NS with deafness as the main clinical feature (Huang et al., 2019). Wu Jie used targeted genome sequencing to make molecular diagnosis for a large number of hearing loss people (Wu et al., 2022). Mutai Hideki analyzed the whole exome analysis of Japanese hearing loss patients, which showed a high degree of heterogeneity between responsible and new candidate genes (Mutai et al., 2022). Cesca Federica analysised of the frequency of Usher gene mutations in non-syndromic hearing loss (Cesca et al., 2020). These studies on deafness have not yet involved PTPN11 gene mutation syndrome.

There are also many researches on deep learning in the field of deafness. Bing D adopted the prognosis classification of sudden deafness based on deep learning method (Bing et al., 2018). Huang Xin used machine learning and deep learning methods to predict the ototoxicity caused by drugs in silicon (Huang et al., 2021). Waldo Nogueira remixed the cochlear implant users with the deep learning model (Gajęcki and Nogueira, 2018). However, there are few researches on the diagnosis and gene function of PTPN11 gene mutation syndrome deafness in the field of deep learning in deafness disease.

In order to enhance the public’s understanding of comprehensive deafness, this paper analyzed the data of 85 deaf children who visited Hunan Children’s Hospital, P.R. China from January 2020 to December 2021, and analyzed the patient’s age, gender, hearing loss, imaging examination, deafness gene and number of NS patients. Finally, the feasibility conclusion was drawn.

## 2 Investigation of comprehensive deafness

**FIGURE 1:**
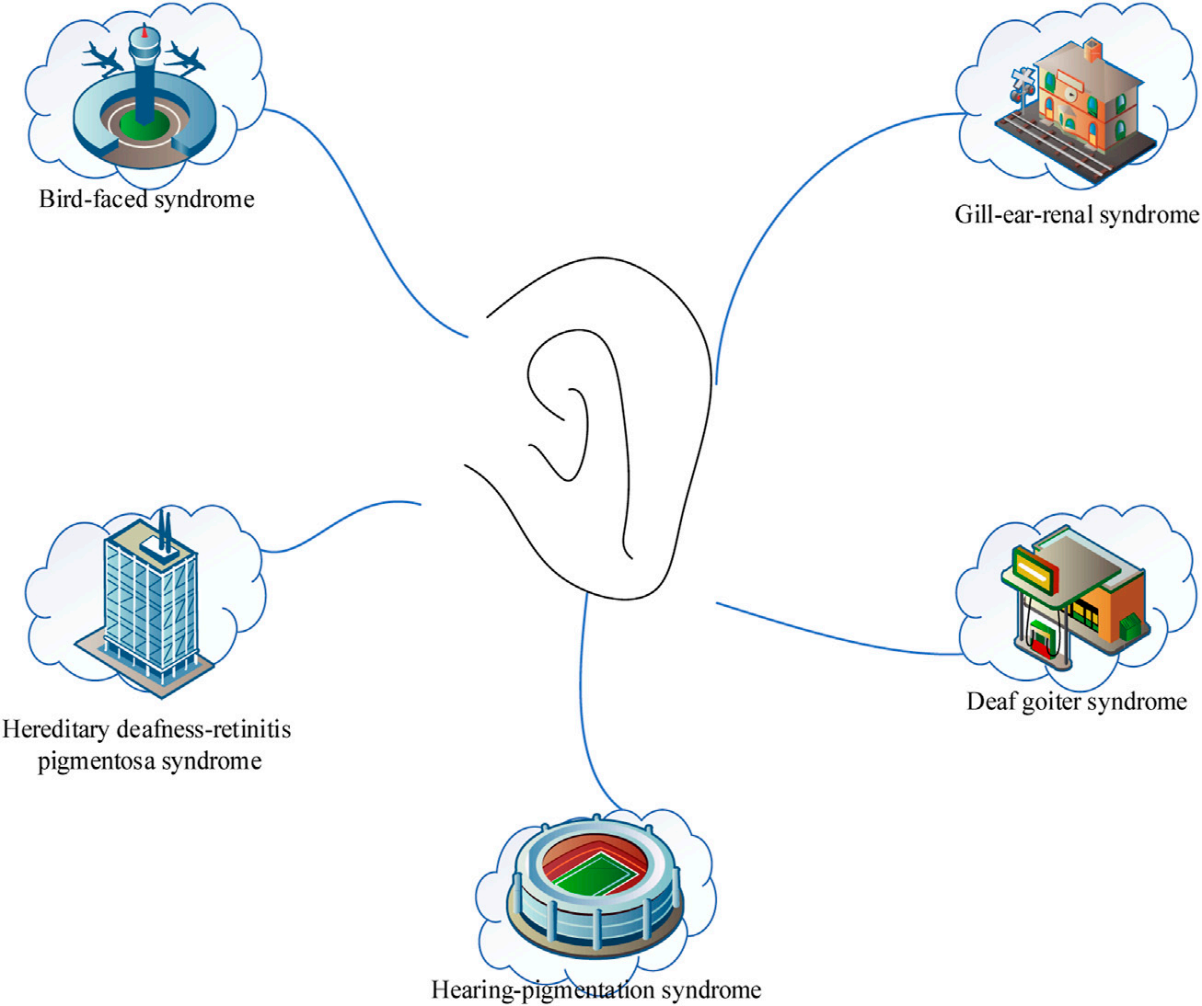
Common comprehensive deafness.

Deafness is a common disease that affects health and human normal life (KimOh et al., 2020), which affects 1%–3% of newborns. Deafness can be divided into two categories: syndromic deafness and non-syndromic deafness. Non-syndromic deafness is mainly caused by genetic deafness caused by deafness gene mutation. About 70% are non-syndromic deafness, and the remaining 30% are syndromic deafness. Syndromic deafness refers to the problem of other organs of the body besides hearing loss. Common comprehensive deafness includes the following types, as shown in Figure 1:

### 2.1 Bird face syndrome

Bird face syndrome is a syndrome of mandibular dysplasia and deafness caused by embryonic agenesis of the first and second branchial arches (Barsottini et al., 2019). The syndrome is characterized by drooping eyelids, hypoplasia of facial bones (especially mandible and zygoma), malformations of the outer and middle ears, and conductive deafness. The vast majority of cases are autosomal dominant inheritance, and up to 70% of cases are sporadic cases. There is no clear family history, but married patients with children have a 53% chance of their children suffering from this disease.

### 2.2 Hereditary deafness-retinitis pigmentosa syndrome

Hereditary deafness-retinitis pigmentosa syndrome is a hereditary disease characterized by congenital sensorineural hearing loss and progressive retinitis pigmentosa (usually from late childhood to adolescence), resulting in visual loss (Deep et al., 2021). The typical clinical symptoms of hereditary deafness and retinitis syndrome are hearing loss and retinitis pigmentosa. Hereditary deafness and retinitis syndrome can be divided into three types according to the degree of hearing loss and the incidence and degree of vision loss. Among them, type I and type II are the most common (about 34%–44% respectively), while type III is relatively rare (12%–32%). The clinical features of hereditary deafness-retinitis pigmentosa syndrome type I include severe congenital deafness, language disorder, vestibular loss or abnormality. The onset age is less than 10 years old, and sometimes there are ataxia, intellectual disability and pseudopsychosis. Hereditary deafness retinitis pigmentosa syndrome type II is characterized by moderate congenital hearing loss and normal vestibular function. This often occurs between the ages of 20 and 30, without language disorders, ataxia, mental retardation or psychosis.

### 2.3 Hearing-pigment syndrome

Hearing-pigment syndrome is characterized by sensorineural hearing loss and abnormal pigmentation (abnormal pigmentation of eyes, skin and hair) (Taneja, 2020). The syndrome is also characterized by abnormal development of the middle nose and forehead, including ectopia of the inner canthus (wide eye distance), eyebrows, broad bridge of the nose and other abnormalities of the middle nose and forehead, as well as skeletal muscle defects in the limbs and congenital megacolon.

Clinical manifestations include inner ear, iris, skin pigment, hair pigment, epicanthus, limb skeletal muscle dysplasia and megacolon. Among them, the inner ear is deformed, and vestibular cistern and semicircular canal are the most common malformations. Some patients may also have cochlear malformations, which are characterized by flattening and slight bending of the cochlea. Skin pigmentation is defined as a large number of brown freckles on the face, neck and/or trunk and limbs. In some cases, there are local white patches on the skin. Hair pigment disorder is manifested as white hair on the forehead or less white hair, which is also manifested as premature lightening of eyebrows and body hair. Ectopia of inner canthus is characterized by wide eye distance, which may be accompanied by abnormalities in the middle of nose and forehead, such as eyebrow linking, broad bridge of nose, etc. Abnormalities of skeletal muscles of the extremities are manifested in muscular dysplasia of the extremities and contracture of the elbow joint (finger joint). Hirschsprung’s disease is congenital Hirschsprung’s disease or gastrointestinal atresia.

### 2.4 Deafness goiter syndrome

Most patients with deafness and thigh syndrome have severe to extreme hearing loss at birth (Kral and Sato., 2020). In a few cases, hearing loss occurs in the first few years after birth. After a cold or serious physical shock, hearing loss develops from serious to deep. Goiter is an important feature of deaf goiter. About 80% of patients with deaf goiter syndrome have goiter. It usually occurs in adolescence and is most severe between the ages of 21 and 31. The size of the goiter is not related to the degree of hearing loss.

### 2.5 Gill-ear-kidney syndrome

Gill-ear-kidneysyndrome, also known as hereditary progressive nephritis, is characterized by hematuria, proteinuria and progressive hypoalgesia. Some patients may have extrarenal manifestations, such as hearing loss, eye abnormalities, and tumors of esophageal smooth muscle.

## 3 PTPN11 gene and Noonan Syndrome

PTPN11 gene encodes Src homology two domain-containing protein tyrosine phosphatase (SHP2), which is located on chromosome 12 (12q24). It is expressed in most fetal and adult tissues and plays a key role in cell proliferation, differentiation, survival and death. Diseases associated with mutations in PTPN11 include NS and Noonan syndrome with multiple lenses (NS-ML). NS is a disease with autosomal dominant inheritance. Typical cases are between 1/2,500 and 1/1,000. Both men and women can occur, which often has a family history. In the neonatal period, the main manifestation is the appearance abnormality: The head circumference is large, and the face is small. The forehead is high, and the hair is sparse; the distance between the eyes is large, and the eyeballs are prominent. The inner eye is ridge, and the upper eyelid is drooping. The cleft palate is horizontal or downward inclined; the nose is short, and the bridge of the nose is low. The tip of the nose is full; the ear is low and the earlobe is oval; the midline is deep, and the upper lip is full. The upper jaw is high, and the lower jaw is small; the neck is short, and the neck is arched. The skin is loose, and the hair line on the back is low.

PTPN11 is the first known pathogenic gene and the most common NS gene, accounting for about 50% of all cases. SHP2 is a protein product of PTPN11 and a non-receptor protein tyrosine phosphatase, which is composed of the N and C terminal domains of SH2 and the catalytic domain of protein tyrosine phosphatase. Most mutations of PTPN11 leading to NS are located in residues related to the interaction between the N-terminal SH2 domain of SHP-2 and the domain of protein tyrosine phosphatase. Mutations in this region destroy the stability of the catalytic inactive form of SHP2 and the ability of the protein to switch from the active conformation to the inactive conformation, which leads to increased signal transmission through the RAS-MAPK pathway.

SHP2 is involved in the regulation of RAS-MAPK signal pathway and the expression of various biological factors and cytokines, such as growth hormone, insulin-like growth factor and fibroblast growth factor. These factors cause various clinical symptoms in children with CF, including growth retardation and bone deformity. Patients with PTPN11 mutation are more likely to have short stature, thoracic malformation, cryptorchidism and mild bruising than those without PTPN11 mutation. N-methyl-d-aspartate receptors are critical for synaptic plasticity, learning, and memory. Tyrosine phosphorylation of N-methyl-d-aspartate receptor subunit regulates the function of N-methyl-d-aspartate receptor. SHP2 dephosphorylates SN related glutamate receptors, thereby regulating the function of N-methyl-d-aspartate receptors and leading to cognitive impairment of SN. Imaging showed that the volume of gray matter on both sides of the striatum decreased, and the area of the temporal region decreased. The thickness of the frontal cortex increased, while the thickness of the marginal cortex decreased, including the marginal structures that are important to the hippocampus. Hippocampus affects the attention and memory during the development of human brain, which is the expression of severe attention deficit, hyperactivity and memory disorder. The existence of PTPN11 mutation in NS patients is closely related to their hematoma susceptibility. The disorder of SHP2 peroxidase would increase the adverse reaction of platelets and the phenotypic coagulation dysfunction. The activated PTPN11 mutation plays a leukemic role in the stem cell microenvironment. Therefore, the over activation of interleukin 1β and other proinflammatory cytokines that can be produced by hematopoietic stem cell monocytes leads to the deterioration of myeloproliferative tumors and donor cell mediated myeloproliferative tumors in children after stem cell transplantation.

## 4 Common molecular diagnostic techniques for deafness

**FIGURE 2:**
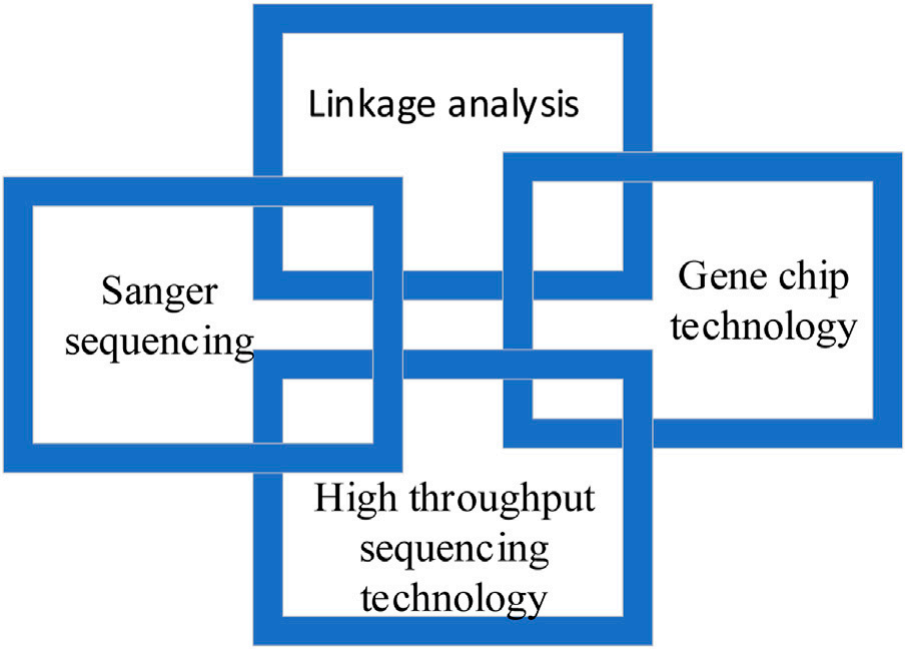
Common molecular diagnostic techniques for deafness.

Common molecular diagnosis technologies for deafness include linkage analysis, gene chip technology, Sanger sequencing and high-throughput sequencing technology, as shown in Figure 2.

### 4.1 Linkage analysis

Linkage analysis is a classical method to study single gene disease. Genes are arranged linearly on chromosomes, while linkage analysis is based on the principle that different genes are linked in linkage groups. That is to say, the gene of interest is linked with another gene or genetic marker on the same chromosome, so as to find the disease causing gene. However, linkage analysis has some limitations. One is that it is not suitable for finding mutations in small families, because mutations in small families are difficult to separate. The other is that linkage analysis usually involves millions of chromosomes containing hundreds of genes and base pairs, which cannot be used to detect diseases. In order to remedy the defects of linkage analysis, gene chip technology is developed.

### 4.2 Gene chip technology

In recent years, gene chip technology has developed rapidly, which is not only limited to DNA detection, but also widely used in non-coding RNA, expression profiling, methylation and other fields. In the field of hereditary hearing loss, the chip hybridization of oligoribonucleic acid is usually used to detect hearing loss genes in the population in clinical practice, and the three-step method of multiple PCR amplification, chip hybridization and result digitization is used. Due to the high throughput, accuracy and low cost, gene chip technology for deafness is widely used in clinical practice.

### 4.3 Sanger sequencing

Sanger sequencing is also known as “first generation sequencing”. It uses DNA polymerase to lengthen the primers attached to the template sequence until the fluorescent labeled dideoxynucleotides enter the polymerization reaction, so as to stop the polymerization reaction and produce DNA fragments of different lengths. Sanger sequencing has the advantage of high precision, but it is gradually being replaced by high-throughput sequencing due to its short reading time, high cost and low throughput.

### 4.4 High throughput sequencing technology

High throughput sequencing technology is also known as “next-generation”sequencing technology. This is an important milestone of human genomics, which has reduced the cost and time of human genome design and is now widely used in clinical and scientific research.

## 5 Application of deep learning and intelligent medicine in medicine

### 5.1 Deep learning

At present, deep learning technology has been applied in many fields, and gradually developed from the initial simple applications such as processing still images and filling in text information to large-scale applications such as processing video and audio data and from incomplete information to creative work. Based on big data, artificial intelligence can quickly deduce the general laws of underlying data processing and preprocessing methods by analyzing previous data and preprocessing methods, and can process and integrate these laws. It can also learn the processing principles, and create new work on this basis. The surface creative activities include painting, writing, language production, music creation, etc. High level activities may include intelligent management, situation assessment, strategic development, etc.

Deep learning is used to solve many problems with big data, such as computer vision, pattern recognition and natural language processing. Artificial intelligence technology based on deep learning is being integrated into clinical practice and medical care. At present, it is widely used in automatic medical image analysis and auxiliary diagnosis, such as classification of benign and malignant lung tumors and detection of benign and malignant breast lesions. It is also analyzing and testing bone, cartilage and other structures in the musculoskeletal field. The accuracy of diagnosis can be improved through the joint analysis of several test equipment; even electrocardiogram analysis can supplement the diagnosis of dysfunction, such as arrhythmia and cognitive impairment, by analyzing arrhythmia, cognitive impairment and other health problems.

### 5.2 Intelligent medical treatment

Smart medicine is to realize the interconnection between patients and medical personnel, medical facilities and medical equipment by establishing the medical records of the regional health information platform and using the latest deep learning technology, so as to gradually realize continuous informatization (Srinivasa et al., 2018). In the near future, more and more artificial intelligence, sensor technology and other advanced technologies would be introduced into the medical and health industry, which would make medical and healthcare become truly intelligent and contribute to the successful development of medical and health undertakings.

#### 5.2.1 Medical equipment and drug information query

The barcode or two-dimensional code on the medical device or package is printed by the laser printer, so that the purchaser can easily check the authenticity of the medical device and drugs, and understand the use of the medical device and the recommended manufacturer, so as to obtain drug information, product, date, and series, and query drug sales information (Gopal et al., 2019; Sodhro et al., 2020). This information can effectively help buyers understand medical equipment.

#### 5.2.2 Monitoring of medical waste disposal

Medical waste is different from ordinary waste, so the treatment of medical waste has a complete process: Deep learning technology can track the process of medical waste from transportation to treatment in real time, which effectively prevents violations in medical waste treatment.

#### 5.2.3 Digitization of medical information

Deep learning technology can also promote the rapid development of mobile medicine. With intelligent medical equipment, people’s important health indicators can be regularly uploaded to the medical information center. The information center can establish a health map for everyone, and monitor people’s health indicators, so as to report people’s physical conditions through mobile communication and provide relevant suggestions on people’s lifestyle, nutrition, etc.

#### 5.2.4 Telemedicine

Deep learning technology can upload patient information from different regions or even countries to the data center. For some severe patients or complex cases, authorized expert advisory teams can provide remote or virtual consultation, so that patients can receive timely, advanced and scientific treatment. In rural areas with low level of medical care and infrastructure, telemedicine based on deep learning technology can improve the level of rural medical care.

## 6 Experimental evaluation of gene function of deafness caused by PTPN11 gene mutation syndrome

### 6.1 General information of the research object

At present, most of the deaf patients with PTPN11 gene mutation syndrome are young children, so this paper conducted a retrospective analysis of the clinical data of 85 deaf children who visited Hunan Children’s Hospital,P.R. China from January 2020 to December 2021. All subjects received detailed medical history and general physical examination, as well as systematic hearing test, inner ear X-ray examination and coronal thin slice computed tomography, so as to exclude hearing loss caused by unilateral deafness and otitis media, meningitis, occupational injury and trauma. After these comprehensive assessments, the study participants were diagnosed with bilateral sensorineural hearing loss.

### 6.2 Hearing assessment of patients

Listening assessment is based on the principles of test matching and cross validation. Auditory physiology and behavioral audiometry were conducted, namely, brainstem evoked response, stable auditory evoked response, paradoxical otoacoustic emission, acoustic conductance and behavioral audiometry. The results were confirmed at the end of the auditory evaluation, and there was a good correlation between the test and retest. The hearing loss of children under 2 years old is determined according to the threshold of V wave response, and the hearing loss of children over 2 years old is determined according to the average of the four frequencies (500 Hz, 1,000 Hz, 2,000 Hz, and 4,000 Hz) felt in the behavioral audiometry test (Shoham et al., 2019). The assessment principles for hearing loss of children under 2 years old can be shown in Table 1.

**TABLE 1:** Hearing loss in children under 2 years of age level of hearing loss.

Sound intensity	Level of hearing impairment
≤ 30dBnHL	Normal
31–50dBnHL	Mild hearing loss
51–70dBnHL	Moderate hearing loss
71–90dBnHL	Severe hearing loss
>90dBnHL	Very severe hearing loss

The assessment principles for hearing loss of children over 2 years old can be shown in Table 2.

**TABLE 2:** Hearing loss in children over 2 years of age level of hearing loss.

Sound intensity	Level of hearing impairment
≤ 25dB HL	Normal
25–40dB HL	Mild hearing loss
41–60dB HL	Moderate hearing loss
61–80dB HL	Severe hearing loss
> 80dBHL	Very severe hearing loss

### 6.3 Methods of gene detection

Genomic DNA is extracted from peripheral venous blood of children and their parents, and high-throughput full exome sequencing is performed using Illumina NovaSeq6000 sequencing platform. Under the 150 PE sequencing mode, Agilent V6 chip is used to extract about 20,000 genes from the human genome and enrich them in the exon region and adjacent transverse regions. At least 10 Gb–12 Gb of data is generated from each sample.

The criteria for evaluating NS are shown in Table 3.

**TABLE 3:** Diagnostic criteria for Noonan syndrome.

Clinical features	Major	Secondary
Facial	Typical face	Suggestive face
Cardiac	Pulmonary stenosis and/or ECG abnormalities	Other abnormalities
Height	< P3	< P10
Chest wall	Pectus excavatum or funnel chest	Thoracic widening
First degree relatives	Confirmed diagnosis of Noonan’s syndrome	Prompt Noonan syndrome
Other	Intellectual disability, cryptorchidism and lymphatic duct dysplasia present together	Intellectual disability, cryptorchidism and lymphatic duct dysplasia

### 6.4 Experimental results of gene function in deafness with PTPN11 gene mutation syndrome

#### 6.4.1 Patient’s age

**FIGURE 3:**
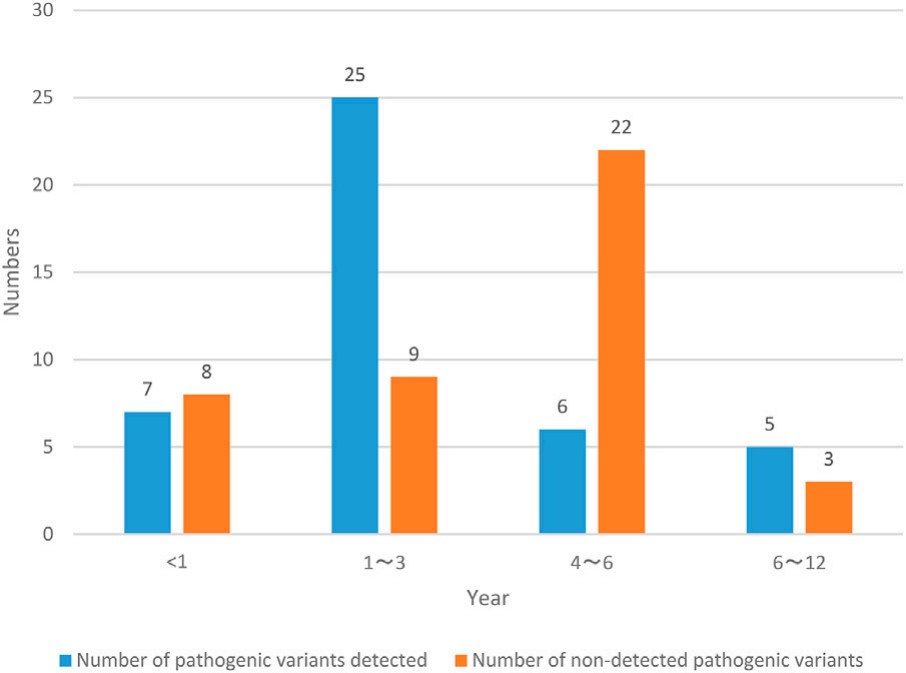
Age profile of patients.

NS patients are divided into two groups according to the number of pathogenic variants detected and the number of pathogenic variants not detected, and the age status of NS patients is recorded in Figure 3.

The NS patients investigated in this paper were all 12 years old and below. Among them, the number of NS patients less than 1 year old detected pathogenic variants was 7, with a probability of 8.24% (7/85); the number of NS patients in the age range of 1–3 years old was 25, with a probability of 29.41% (25/85); the number of NS patients in the age range of 4–6 years old was 6, with a probability of 7.06% (6/85); the number of NS patients aged 6–12 years old with pathogenic variants was 5, with a probability of 5.88% (5/85); the number of NS patients less than 1 year old without pathogenic variation was 8, with a probability of 9.41% (8/85); the number of NS patients aged 1–3 years without pathogenic variation was 9, with a probability of 10.59% (9/85); the number of NS patients aged 4–6 years without pathogenic variation was 22, with a probability of 25.88% (22/85); the number of NS patients aged 6–12 years old with pathogenic variants was 3, with a probability of 3.53% (3/85). The number of NS patients aged from 1 to 3 years with pathogenic variation detected and the number of NS patients aged from 4 to 6 years without pathogenic variation detected are the largest, and the probability is also high.

#### 6.4.2 Patient’s gender

**FIGURE 4:**
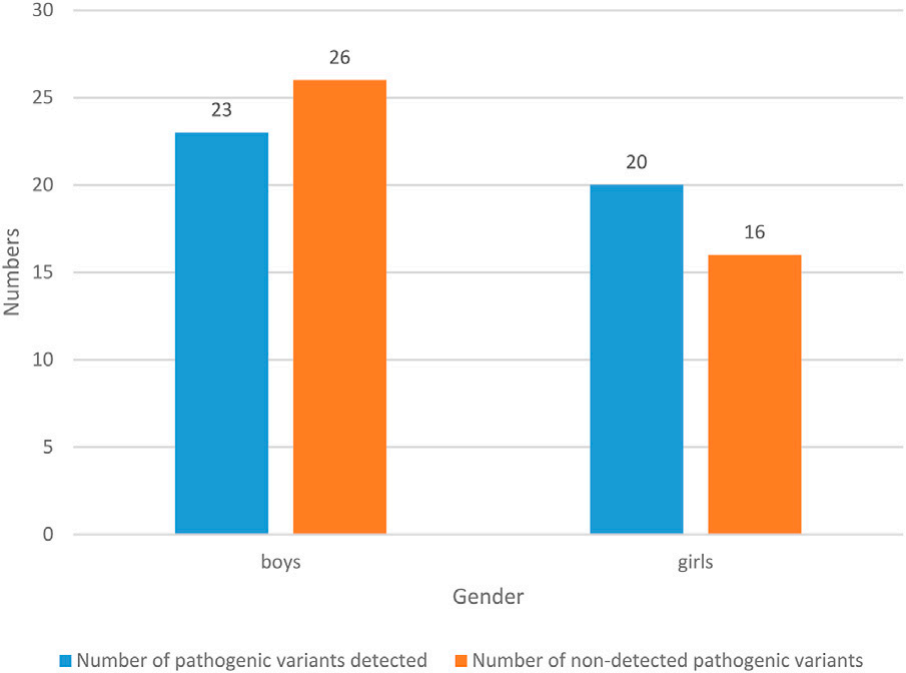
Gender status of patients.

The results of gender status of NS patients are shown in Figure 4.

49 NS children were boys, with a probability of 57.65% (49/85). Among them, the number of pathogenic variants detected was 23, with a probability of 27.06% (23/85); the number of people without pathogenic variation was 26, with a probability of 30.59% (26/85). 36 NS children were girls, with a probability of 42.35% (36/85). Among them, the number of pathogenic variants detected was 20, with a probability of 23.53% (20/85); the number of pathogenic variants detected was 16, with a probability of 18.82% (16/85). In general, boys are prone to NS, and the probability of detecting pathogenic variants is lower than that of not detecting pathogenic variants.

#### 6.4.3 Hearing loss of patients

**FIGURE 5:**
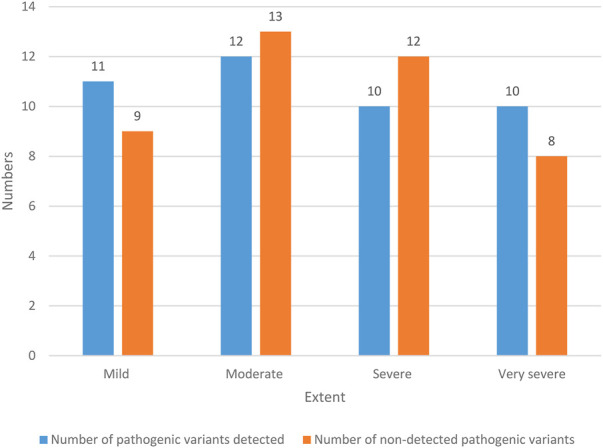
Hearing impairment of patients.

The hearing loss of NS patients is shown in Figure 5.

There were 20 NS patients with mild hearing loss, and the probability was 23.53% (20/85). Among them, 11 NS patients with mild hearing impairment detected pathogenic variation, with a probability of 12.94% (11/85); there were nine patients with mild hearing impairment NS who had no pathogenic variation, with a probability of 10.59% (9/85). There were 25 NS patients with moderate hearing loss, with a probability of 29.41% (25/85). Among them, 12 NS patients with moderate hearing loss detected pathogenic variation, with a probability of 14.12% (12/85). There were 13 NS patients with moderate hearing loss who had no pathogenic variation, with a probability of 15.29% (13/85). There were 22 NS patients with severe hearing loss, with a probability of 25.88% (22/85). Among them, 10 NS patients with severe hearing impairment had pathogenic variants, with a probability of 11.76% (10/85). There were 12 NS patients with severe hearing impairment who had no pathogenic variation, with a probability of 14.12% (12/85). There were 18 NS patients with extremely severe hearing loss, with a probability of 21.18% (18/85). Among them, 10 patients with extremely severe hearing impairment NS had pathogenic variants, with a probability of 11.76% (10/85). There were 8 NS patients with extremely severe hearing impairment who had no pathogenic variation, with a probability of 9.42% (8/85). In general, the hearing loss of NS patients is mostly moderate and severe.

#### 6.4.4 Imaging examination

**FIGURE 6:**
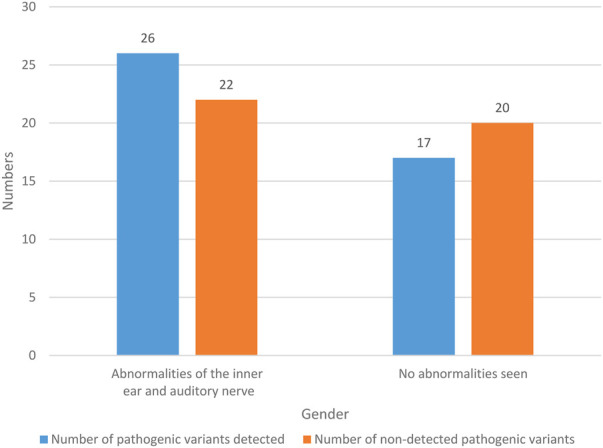
Imaging studies.

The imaging findings of 85 NS patients are recorded in Figure 6.

The imaging findings of 48 NS patients were abnormal in the inner ear and auditory nerve, with a probability of 56.47% (48/85). Among them, pathogenic variants were detected in 26 NS patients with abnormal inner ear and auditory nerve, with a probability of 30.59% (26/85). There were 22 NS patients with abnormal inner ear and auditory nerve, and no pathogenic variation was detected, with a probability of 25.88% (22/85). There were 37 NS patients with no abnormal imaging results, and the probability was 43.53% (37/85). In these NS patients, pathogenic variants were detected in 17 patients, with a probability of 20% (17/85). There were 20 patients without pathogenic variation, and the probability was 23.53% (20/85). It can be seen that NS patients often have abnormal inner ear and auditory nerve.

#### 6.4.5 Deafness gene and number of cases in NS patients

The deafness genes and cases of 85 NS patients are recorded in Table 4.

**TABLE 4:** Deafness causative genes and number of cases in NS patients.

Gene	Type of mutation	Mutation location	Nucleotide variant	Amino acid variant	Source	Number
PTPN11	Missense mutation	Exon13	c.1510A>G	p.M504V	Spontaneous	15
PTPN11	Missense mutation	Exon1	c.10C>G	p.R4G	Spontaneous	24
PTPN11	Missense mutation	Exon3	c.236A>G	p.Q79R	Spontaneous	25
PTPN11	Missense mutation	Exon11	c.1277A>G	p.H426R	Spontaneous	21

Among 85 NS children with PTPN11 gene mutation, 15 children had a gene mutation of Exon13, and the nucleotide mutation was c.1510A>G. The amino acid variation was p.M504V, with a probability of 17.65% (15/85); there were 24 children with Exon1 gene mutation, and the nucleotide mutation was c.10C>G. The amino acid variation was p.R4G, with a probability of 28.24% (24/85); the gene mutation of 25 children was Exon3, and the nucleotide variation was c.236A>G. The variation of amino acid was p.Q79R, and the probability was 29.41% (25/85); the gene mutation position of 21 children was Exon11, and the nucleotide variation was c.1277A>G. The amino acid variation was p.H426R, with a probability of 24.7% (21/85). In general, most of the mutation genes in children are located in Exon1 and Exon3, with a combined probability of 57.65%.

## 7 Conclusion

In order to study the diagnosis and gene function of deafness caused by PTPN11 gene mutation syndrome, this paper first introduced the syndrome deafness, and then analyzed the PTPN11 gene and Noonan syndrome. The common molecular diagnostic techniques for deafness were introduced, and the application of deep learning and intelligent medicine in medical treatment was analyzed. Finally, the gene function of deafness caused by PTPN11 gene mutation syndrome was analyzed, and the conclusion was drawn. The number of NS patients aged from 1 to 3 years with pathogenic variants detected and the number of NS patients aged from 4 to 6 years without pathogenic variants detected were the largest. Boys were prone to NS, and the probability of the number of people with pathogenic variants detected was lower than that of the number of people without pathogenic variants detected. The hearing loss of NS patients mostly showed moderate and severe damage, and NS patients often had abnormal inner ear and auditory nerve. Most of the mutation genes of the children were located in Exon1 and Exon3. Deep learning technology had an excellent application in analyzing the gene of NS patients.

## Data Availability

The original contributions presented in the study are included in the article/Supplementary Material, further inquiries can be directed to the corresponding author.
